# Toward Understanding How Social Factors Shaped a Behavioral Intervention on Healthier Infant Formula-Feeding

**DOI:** 10.1177/1049732318764386

**Published:** 2018-03-21

**Authors:** Cornelia Guell, Fiona Whittle, Ken K. Ong, Rajalakshmi Lakshman

**Affiliations:** 1 University of Exeter, Truro, UK; 2 University of Cambridge, Cambridge, UK

**Keywords:** behavior change, complexity, diet, nutrition, malnutrition, mothers, mothering, infants, obesity, overweight, stigma, Britain, qualitative methods, qualitative

## Abstract

As part of a process evaluation, we explored in semi-structured interviews the experiences of 19 mothers who had taken part in a trial to reduce infant formula-milk intake and promote healthy weight gain, and reflections of three facilitators who delivered the intervention and control group protocols. Mothers appreciated the nonjudgmental support provided during the trial, after experiencing stigma and receiving limited advice on how, how much, and how often formula-milk should be given. The information mothers had previously found, printed on formula-milk tins, or provided by family, friends, and health professionals was often perceived as contradictory; the trial guidance also conflicted with social norms relating infant health positively with weight gain. For those recruited into the trial with older infants, who had already exceeded the recommendations, reducing formula-milk amounts was difficult. The findings highlight the difficulties of addressing a highly stigmatized, complex social practice with an individual, behavioral intervention approach.

Childhood obesity is a major public health challenge (World Health Organization [WHO], 2016). Efforts to prevent obesity in later years of childhood and adolescence are considered less effective than early intervention in infant years (Taveras, 2016), so one focus has been on infant formula-feeding as it is associated with rapid excess weight gain and later obesity (Ong & Loos, 2006). Rates of exclusive breastfeeding below 6 months and continued breastfeeding at 12 months of the infant’s age are still relatively low in the United Kingdom (Victora et al., 2016). This is despite widespread knowledge of its benefit for infants’ health and concerted efforts such as the UNICEF Baby Friendly Initiative to support the initiation and continuation of breastfeeding (Pound & Unger, 2012).

Considering that most infants receive some formula-milk, it is therefore important to also support parents to feed healthy amounts. In 2004, the WHO reduced the recommended energy requirements for infants, by 15% to 20%, in response to new data on energy expenditure (Food and Agriculture Organization, WHO, & United Nations University, 2004). As many infants exceed this guidance leading to rapid infant weight gain (Ong, Emmett, Noble, Ness, & Dunger, 2006), the *Baby-Milk Trial* aimed to evaluate the efficacy, cost-effectiveness, and acceptability of a theory-based, multicomponent behavioral intervention to reduce formula-milk intake based on the new WHO recommendations and prevent excess weight gain during infancy (Lakshman et al., 2015).

Previous research on infant formula-feeding tended to focus on understanding the barriers to and facilitators of taking up or continuing breastfeeding (Brouwer, Drummond, & Willis, 2012; Mannion, Hobbs, McDonald, & Tough, 2013), or the experience of breastfeeding itself (Ryan, Todres, & Alexander, 2011). Studies on understanding formula-feeding tend to focus on decision making (over breastfeeding; Hoddinott, Craig, Britten, & McInnes, 2012; Sheehan, Schmied, & Barclay, 2013) or the moral discourse surrounding formula-feeding (Lee, 2007). Very little research has been conducted on exploring the actual practice of formula-feeding itself. As part of the intervention development of the *Baby-Milk Trial*, a review of the literature and a qualitative study with mothers and health care professionals found that mothers preferred general guidelines to rigid rules, did not believe that infants could be overfed, and considered obesity prevention for infants as too early (Lakshman et al., 2012; Lakshman, Ogilvie, & Ong, 2009; Lee, 2007). The *Baby-Milk Trial*’s intervention set out to educate new mothers about healthy ways of formula-feeding.

The qualitative study, reported in this article, was conducted toward the end of the *Baby-Milk Trial*, and followed the objectives of qualitative process evaluation of complex interventions to use qualitative methods to examine whether the intervention was delivered as intended, to unpack processes of implementation and behavior change, and to explore the facilitators’ and recipients’ responses to and experiences of delivering and taking part in the trial (Moore et al., 2015). This article aims to explain some of the underlying mechanisms that might have been at play when implementing and participating in the *Baby-Milk Trial* and shaped its outcome.

## Method

### The Baby-Milk Trial’s Theoretical Framing and Process

The trial’s intervention component had been developed to teach new mothers about healthy ways of formula-feeding and contained a motivational component and an action-planning component to support parents to follow the *trial’s* feeding guidelines, and a coping-planning component to help parents deal with difficult situations (Lakshman et al., 2015). Based on Social Cognitive Theory, the emphasis was on improving parents’ self-efficacy in healthy feeding practices, influencing their outcome expectancy so they understand the benefits of the feeding guidelines, and thus strengthening their intentions and motivations. As trial participants (intervention and attention control), the mothers received five face-to-face visits and phone calls in their infants’ first 6 to 7 months, with the option to get in touch (by email or phone) with the facilitator with additional questions. In addition, their infants were regularly monitored for growth.

### This Qualitative Study’s Theoretical Framing

We deliberately did not base the analysis of this qualitative evaluation on the trial’s theoretical framing in Social Cognitive Theory or other behavior change frameworks but aimed to approach these women’s experiences more inductively and holistically. However, as the women’s experience seemed strongly interlinked with others (the intervention facilitators, family, other health professionals), the analysis was theoretically guided by social practice theories that aim to understand in what way everyday practices such as infant feeding are socially shared and shaped through time and space (Maller, 2015; Reckwitz, 2002; Southerton & Welch, 2015).

### Participants

Ten mothers from the intervention arm of the trial, and nine from the control group, as well as three of the four female facilitators (research nurses) took part in this qualitative study. It seemed important to understand the experiences of the women who got explicit guidance on healthy strategies for formula-feeding in the intervention arm, as well as of those mothers in the attention control group who received the same amount of contacts but only general guidance such as sterilizing bottles, feed preparation, and types of formula-milk. Mothers were recruited from the last wave of families that had just completed the trial protocol from an eligible pool of 58 trial participants, of whom 43 were contacted (in the first instance, and the response deemed sufficient for a relatively homogeneous sample) and invited to this qualitative study, and of whom 19 took part (44% response rate). The participants had been initially recruited to the trial if identified as formula-feeding by research staff or health professionals (Whittle, Ong, Griffin, & Lakshman, 2015). Our study participants’ infant’s age at the time of recruitment into the trial was between 3 and 14 weeks, with an average age of 8 weeks (10 weeks for the overall trial). These infants were on average 7 months old by the time their mother was interviewed for this qualitative study. For 11 of the 19 women, this was their first child. We included the voices of the facilitators to provide us with their reflections on delivering the intervention and control protocols, and their positive and negative experiences.

### Data Collection

Between July and October 2015, 22 semi-structured interviews were conducted in person at a place of the participants’ convenience (mostly at home, mostly with their infants present; and the facilitator interviews conducted at their workplace). Interviews were conducted on average one month after the intervention ended at the infant’s age of 7 months. The interviews with the mothers were conducted by a female member of the trial team who was the trial manager but had no previous personal contact with mothers before the interviews. The interviews with the facilitators were conducted by a female researcher outside the trial team who had not met the facilitators before. Interviews lasted between 32 and 77 minutes, and all were audio-recorded and transcribed verbatim. Interview questions (see Online Supplementary File 1) included the women’s reasons for formula-feeding, their experience of formula-feeding prior to and during the trial, and their experiences and reflections on participating in the trial. The facilitators were also asked to comment on these issues as they had learned from the women during the trial, as well as their own reflections on the trial design and delivering the intervention and control group protocols.

### Data Analysis

We conducted thematic analysis. We double-coded sample transcripts from participants representing both the control and intervention groups, then iteratively developed a coding tree that covered categories of trial participation, feeding experience, support and information, and stigma. With the finalized code book (see Online Supplementary File 2), the rest of the transcripts were coded. Two researchers identified an initial set of themes before the outcome of the trial was known; a second wave of analysis together with the rest of the research team explicitly explored potential explanations of the trial findings. Particular attention was placed on constant comparison between cases (intervention and control participants, and facilitator and participant interviews) and on negative cases to ensure rigor in the analysis. All participants, including the facilitators, gave their informed written consent for this qualitative study separate from trial ethical procedure. Ethical approval was granted by Cambridge South Research Ethics Committee (10/H0305/9).

## Results

We identified three themes that describe the women’s experience of taking part in a formula-feeding trial. Theme 1 shows that the mothers we interviewed felt that they lacked support for bottle-feeding their infants, particularly in contrast to the support breastfeeding new mothers seemed to get in their view. Trial participation remedied this gap. Theme 2 explores the way in which the advice and information they did receive outside the trial often seemed contradictory. The trial guidance seemed reliable and clinical measurements reassuring; however, the main trial message introduced similar contradiction, in particular, that weight gain might not be necessarily positive. Finally, Theme 3 describes the added challenge of being required to feed reduced amounts of formula-milk when joining the trial with an older infant.

### Theme 1: Receiving Professional Attention When Support Was Felt Withheld

Most mothers decided to take part in the *Baby-Milk Trial* because they hoped for more support and information on infant formula-feeding. When asked about the positives about the trial, all women mentioned the personalized support and attention they got as the main benefit of the trial, and how much they appreciated it.

Q: How have you found taking part in the study?

A: Really good. Loads of information, I wouldn’t really put any, have anything sort of doubtful to say about it, really. I think [the facilitator]’s been really good, and obviously another girl came with her to do some weigh-ins and stuff occasionally. But, yeah, no, I’ve really enjoyed it, loads of information, a lot of guidance as well, and she made me feel quite sort of happy and content by always making sure, if I needed to call her or anything like that, she was always there, so, yeah. (Intervention Group Participant I)

A: Yeah, very good, very interesting [taking part in the study], I liked it, yeah, because I had the support of [the facilitator] and she said to me “If you need me just send me an email or call me and I will be there,” . . . so yeah, I liked it very much. (Control Group Participant C)

This access to formula-feeding advice and support was in stark contrast to the women’s experience before entering the trial. Before their participation, they recounted relying on sparse information provided on leaflets and formula-milk tins, or advice perceived as reluctantly given by midwives or health visitors. All mothers felt that the health professionals that they encountered outside the trial were not only often too busy to give detailed advice but also reluctant to provide support for infant formula-feeding if this could deter from attempts to breastfeed. The trial facilitators (research nurses and also health professionals), on the contrary, provided lots of advice and time.

Q: . . . what was it that made you decide to take part?

A: Well, . . . it was offering quite a lot of advice that I hadn’t been given by any other health . . . professionals . . . it’s very difficult because you’re led to believe that breastfeeding is best and that’s the way to do it, but obviously from my point-of-view, it didn’t happen, it was bottle-feeding and it was like, “I don’t know how much to give,” when we left the hospital it wasn’t very clear, how often do I do it, do I feed on demand or do I do sort of set feeds, you know, and for a first-time mum, it was just very confusing. To have something there that could offer that sort of support and advice and a bit of guidance, was just great, to be honest. (I)

But no-one gave me anything, I know I didn’t end up bottle-feeding until towards the end but as soon as you go into hospital it’s like breastfeeding-breastfeeding, there’s no advice and especially because of the Baby Friendly Initiative . . . They just don’t provide you with the support . . . you should be supporting those parents regardless of what they choose because it’s the baby’s health and wellbeing. (I)

Mothers not only reported limited support from health professionals outside the trial but also described experiencing negative reactions from others including friends and the public about their infant formula-feeding. In the interviews, mothers recounted frequently needing to justify why their infant was formula-fed, and avoiding such experiences by feeding their infants before going out.

. . . it’s like other parents looking down on you, that are breastfeeding, I found that that was a major thing. If I went to any baby groups, I’d try and make sure that she’d already had a bottle. (I)

I think for, the thing that really frustrates me is that everyone goes on about “breast is best” and then when you bottle-feed they make you feel a bit like you’ve let your baby down, a bit like you’re a failure, and there’s all these like support groups, breastfeeding support groups in the community, . . . and there’s not that for bottle-feeding . . . I’ve had negative comments from some friends who didn’t know the reason behind, I just said “oh I’m bottle-feeding” and it’s like “ooh, that’s why he’s poorly at the moment” . . . they don’t know the story, and then I have to say “well actually I didn’t really have the choice to do that” and they’re like “oh sorry.” (C)

Although they did not explicitly discuss this, part of their appreciation of having been part of the trial could have been that they were recruited into a “legitimate” cohort of mothers (trial participants) where breastfeeding is no longer an option to be considered and bottle-feeding destigmatized. Although this experience of stigma was prevailing in most interviews, it has to be acknowledged that not all participating mothers experienced stigma in their interaction with health professionals and highlighted their positive experience during interviews:

. . . they [the midwives] were so brilliant about it, and even in the hospital, obviously you have to go [outside of the hospital] and get the bits and pieces you need, and we . . . took the stuff [bottles and formula] in, . . . they were absolutely fine, nobody turned their nose up, nobody gave you any dodgy looks, and back when I was younger, people would. (C)

### Theme 2: Receiving Trial Advice on the Backdrop of Disparate, Informal, and Often Conflicting Information

Trial participants in the intervention group were taught about the WHO guidance on healthy formula-milk amounts, to recognize infants’ satiety cues and therefore not force infants to finish the bottle, and recognize that crying was not always due to hunger and therefore try water or a dummy to calm them. These detailed instructions stood in contrast to the limited advice from elsewhere available to mothers. Instead, women recounted how they had used their own experience with previous children, or that of family and friends, to make decisions about how to feed their infants before entering the trial. These shared experiences from other mothers seemed to offer them more tangible and personalized instructions, rather than the impersonal and generic advice that they gained from instructions on the tin or given on leaflets.

In terms of, you know, bottle-feeding and what formulas and how much and all that kind of stuff, I mean the internet really has been a really good source. As I said, friends, my two best friends had exclusively bottle-fed . . . so a combination of the internet and other friends and your gut really. (C)

However, participants did not only rely on informal lay advice from friends and family but also received information from other health professionals not related to the trial, but this advice was perceived to be not consistent. Although some advised them to “stick to the tin [instructions],” others advised to feed in regular intervals, or to feed on demand, “’cos he’s crying he’s hungry”:

. . . this is the problem, I don’t think there’s a great deal of guidance, obviously you get what’s on the tin which tells you a rough idea of how much you should be feeding your baby, but what I got was conflicting information between the health visitor, a midwife and my GP, because I found it really hard to work out “do I stick to the tin” . . . ’cos that’s what my midwife told me to do, “feed him every couple of hours and make sure he doesn’t go no longer than this amount of time,” which was fine in the beginning and then the health visitor comes round “no, no, no, you should feed on demand ’cos he’s crying he’s hungry.” . . . Yeah, you do feel a bit in the dark and you’ve only got what’s, as I say, the guidance on the tin and then guidance from the healthcare professionals that see you, but they don’t always think on the same hymn sheet . . . (C)

. . . from a feeding point of view, from the day we went for the first meeting he was having a lot more milk than he should have had, so [the facilitator] suggested we cut him back just to see, to what they wanted for the intervention group and I cut him back and he had a weight loss so the [regular] health visitor said you’ve got increase his milk up so I ended up increasing and I think at one point he was having 300mls more a day than they recommended. (I)

It appears that this disparate advice often conflicted with each other, and once they entered the *Baby-Milk Trial* also with the intervention guidance. A case in point is health visitors’ focus on tracking infants’ growth by weight. Any downward trajectory seemed to be problematic for health visitors not connected to the trial, and a trial participant explained that her health visitor had not approved of her infant’s weight loss after regulating down her feeding amounts to follow the intervention guidance. Another woman recounted that the facilitator agreed when another health professional queried the lower feeding amount and revised the feeding plan.

. . . when I left hospital with him, he was underweight and the hospital were saying, “You know, you’ve got to feed him on demand” and [the facilitator] was saying to me “Well do you think you can feed him less,” but when I told her what the hospital said, she said “Well, we can’t really ignore the hospital.” (I)

This also fits with some mothers’ responses to equate weight gain as a positive outcome. Mothers commented on the health of their infants in terms of their regular weight gain, or shared in interviews their concerns for their infant’s health when weight dropped or stagnated.

I’m very, yeah, very positive about it, she’s been happy and healthy and been put on weight regularly. (C)

This was for some related to their initial experience of failing to give their infants enough breastmilk—which had been assessed by the weight gained by the infant.

He doesn’t gain weight very quickly, he’s right at the bottom on the percentiles and there was like one week where he lost weight when he was ill and I was really worried about him I was really struggling, I was in a lot of pain and that was the main issue actually, she wasn’t putting on as much weight as they wanted her to and we’d got to kind of a crisis point where . . . I was told by the breastfeeding lady that I needed to give her formula top-ups. (C)

The more thorough measurements provided during the trial, including not just weight but length and head circumference, helped to reassure the mothers in their worry about underfeeding (as much as overfeeding, as intended), and also showed the juxtaposition with health visitors’ focus on weight gain as a measure of health, as a mother happily recounted that her infant was “in proportion, so that was nice because if I was just going to a health visitor . . . I would have just thought he was underweight but he’s not, he’s just small.” (C)

### Theme 3: Joining the Trial With an Older Infant Demanded Too Much Change of Entrenched Practices

Healthy, term infants who were receiving formula-milk within 14 weeks of birth were eligible for the trial. Initial challenges to recruit women to the trial as identified by health visitors (at infant’s age 2 weeks, or 6–8 weeks when they receive the universal mandated health visitor checks) led to the expansion of recruitment through general practitioners (GPs), facilitators at postnatal hospital wards, and via a mail-out using the National Health Service (NHS) database. On average, women entered the trial 10 weeks after their infant was born. The facilitators speculated that recruitment was mainly hampered by women’s reluctance to speak about formula-feeding and worries about being judged.

I think initially, I think why we’ve had trouble recruiting or recruitment has gone quite slowly is because people won’t talk about formula-feeding and therefore, you know, they’re worried about telling their Health Visitor, they’re worried about . . . and so they don’t, they just think oh it’s another person that’s going to give them a hard time maybe. (Facilitator F)

Some mothers expressed regret that they would have preferred entering the trial straight after the birth of their child to get support from the start, or that they wished they could have entered the trial with their first child. This was because they would have appreciated personalized support straight from the start, but some also realized that they were already exceeding the recommended feeding guidance. Decreasing feeding amounts did not seem to be an acceptable option for most participants who had already overfed their infants by the time they entered the trial. The solution was to stick to the feeding amount until the infant had caught up with the trial recommendations.

I think had we been recruited onto the study earlier, then we might not have been in that set routine and I might have been a bit more receptive to changing things. (I)

No, the only thing I thought it could have been, have improved for us is if they’d got to us earlier and I hadn’t already started feeding her too much, then I wouldn’t have had a problem following the guidelines from the beginning, . . . because I’d already started feeding her too much . . . she [my daughter] did not take kindly to being decreased, I think, so we just left it at the same until she caught up with how much it was supposed to be, and then from that point we were on track and we followed the advice completely and it was fine, and it was good, because without that, I would have kept increasing, increasing, increasing. (I)

I mean we were feeding . . . quite a bit over what the [intervention] guidelines said, we were a bit like “oops!” So we did cut it down slightly without too much interference to [the infant], we didn’t stick to that first, I think it was three months we started, we didn’t stick to the guidelines, because he was a big baby, and [the facilitator] had said, you know, as a big baby he might need a bit more, and I was reluctant to drop it right down. (I)

From the facilitators’ perspective, it also made it difficult to introduce feeding amounts that had already been exceeded. They knew that many women felt regret to formula-feed their infants and worried about the health impact for their children; the facilitators were cautious to add another worry—to be overfeeding. Instead, they were keen to support the women in a positive way, not to call out overfeeding in a judgmental way and damage—often quite anxious—mothers’ confidence, “you almost feel that they’re being bruised,” and to establish a good rapport with the mothers.

So, we never forced anything on parents, and I think that was the nice thing with the intervention, nothing was forced upon the parents, but yet they had the information at hand. So if they wanted it they could do it. (F)

. . . certainly the first year I did this job it was getting the balance of trying to encourage them to follow these new guidelines and trying to sort of express how important this issue is with formula-feeding, without adding to their guilt at formula-feeding in the first place. I didn’t want them to go away thinking “oh, it’s just somebody else who’s telling me in a round about way that I’m not doing the best for my baby.” So it was, sometimes I used to come away from the baselines thinking “have I encouraged or pushed this mum enough, could she have done better or have I been too laidback” . . . (F)

I think it’s very important to have a good rapport with the mothers, I think actually they’re quite vulnerable . . . I kind of said to one of my colleagues that you almost feel that they’re being bruised because they’ve tried breastfeeding and it hasn’t worked and then they’re made to feel guilty about that and by the time they get to us you kind of think, you know, they’re almost, they just want someone to listen to them and hear their experiences. So, I think having a non-judgmental approach is paramount. (F)

Although they might have not reinforced the intervention guidance strictly, the facilitators were trained to recognize that it was important to negotiate feeding practices and bring the mothers on board with the intervention guidance. By allowing for this negotiation of the intervention guidelines, however, the facilitators might have inadvertently supported the view that straying from the guidelines was fine, and at times, this might have been perceived that facilitators condoned excess weight gain.

## Discussion

### Principal Findings

All participants in our study reported the trial participation as a positive experience, and this was in contrast to previous negative experiences as formula-feeding mothers. They shared various experiences of not getting help, support, or information about formula-feeding not only because health visitors or midwives would be too busy but also because this could discourage breastfeeding. Nonetheless, the women reported a variety of information sources, which tended to include advice from friends and family, and from health professionals they encountered in the regular health care system. However, this advice was disparate and often considered contradictory. Weight gain seemed mainly discussed as a positive outcome by other health professionals without attention to excess weight gain. For some women, recruitment into the trial when their infant was older meant that women had already exceeded feeding guidelines and reducing feeding amounts were difficult to achieve for both mothers and the facilitators. Mothers’ experiences of stigma exacerbated the facilitators’ challenge to correct feeding practices sensitively.

### Understanding the Trial Outcome

The trial was effective in improving the psychological determinants set out in Social Cognitive Theory that framed the intervention (maternal confidence, intentions, and perceived benefits of following the feeding recommendations) and maternal attitudes to infant feeding and growth. It was also effective in reducing milk intakes (the target behavior) and weight gain in the first 6 months of life (Lakshman et al., 2017). However, no significant difference was found in weight gain at age 12 months between the intervention and control group (the primary outcome) when infants were weaned on to solid foods (Lakshman et al., 2017). This qualitative study can contribute to the process evaluation of the trial and attempt to explain some of the underlying mechanisms that might have been at play in the following specific ways (Moore et al., 2015).

#### Formula-feeding as a stigmatized practice

The results of this study echo previous studies on negative experience of infant formula-feeding mothers (Lakshman et al., 2009) including experience of guilt and stigma (Hoddinott et al., 2012; Lee, 2007); limited access to support and information, and feeling neglected by health professionals (Hoddinott & Pill, 2000); and reliance on commercial guidance (Lakshman et al., 2012). Behavior change models tend to focus on the individual (or how they perceive their social surroundings); missing in the trial’s theoretical underpinning of individuals’ cognitive attitudes and motivations was an account of power and social, collective forces that might have influenced the trial participation and interactions in several ways. Instead, we conceived of the “target behavior” as a social practice that is socially shaped and meaningful, recursive and relational (Bourdieu, 1980; Reckwitz, 2002), which enabled us to trace why the mothers’ responses to the intervention were so tightly linked to their experiences of stigma. Stigma had been experienced in subtle and not so subtle ways (Goffman, 1963/1990), both institutionally with mothers’ perception that advice about infant formula-feeding had been limited if not withheld by some health professionals on the backdrop of fears to discourage breastfeeding, and in social interactions that required justification or management (e.g., feeding at home before leaving the house) in everyday life.

This experience of stigma might have been an underlying barrier to recruitment, and being recruited with older infants, in turn, became a barrier to following the intervention guidance (e.g., requiring to significantly reduce feeding amounts). Furthermore, the intervention facilitators were highly aware that the women felt stigmatized, or at least insecure about formula-feeding, and went cautiously into the trial interactions, emphasizing their supportive, nonjudgmental role within the trial. Getting positive, nonjudgmental attention and recognition from these research nurses stood out as the most positive experience to all women, even for those in the control group who did not receive specific advice on amounts of formula-milk but general advice about sterilizing bottles, feed preparation, and types of formula-milk. This is also reflected in the high trial retention rate, and the high engagement with the intervention. As the interviews with both trial groups showed, feeding was central to their experience of raising their infants, and their own and others’ perceptions of their ability as mothers. However, the women’s anxieties were still present in most interactions, and messages given by the facilitators, for example, on negative outcomes of overfeeding, might not have been firm enough out of concern to worry these new mothers who already displayed anxiety over their infants’ welfare. It might be argued that the intervention facilitators’ role also included providing pastoral care as well as delivering the intervention messages. It seems vital to understand these complex mechanisms underlying both the practice of infant feeding and the practice of participating in an intervention, and exemplifies critiques of the limited ambition of “complex interventions” to truly account for complexity (Cohn, Clinch, Bunn, & Stronge, 2013).

#### Feeding as a complex practice

Although its approach was that of a complex intervention (Lakshman et al., 2015), infant formula-feeding, or feeding more generally, as a complex behavior might have been underestimated beyond the challenge of stigma. As most of the participants of this qualitative study had entered the trial when their infants were older, they had developed formula-feeding practices first without help, and then in reaction to the intervention guidance. The women seemed to have learned self-reliance in their previous experiences, and this might have had a negative impact on the adherence to the intervention when women struggled with the recommendations (for example, when they needed to downregulate formula-milk amounts) and reverted back to their initial instinctive and reactive ways of feeding according to their infants’ demand. The intervention did not necessarily override women’s “what’s best for the baby” practices. A central message of the behavioral intervention was to teach the mothers that they should listen to the infants’ cues. Enhancing self-efficacy to listen to infants’ needs, therefore, might have reinforced entrenched practices developed before the trial.

Moreover, feeding practices had been developed with the help of limited, disparate, and often conflicting advice given outside the intervention from other health professionals, friends and family; perhaps most impactful for the intervention result was a general emphasis of infants’ weight gain as positive without explicit recognition of *excess* weight gain as a problem. Strong societal norms that connect infants’ weight gain with healthy growth seem to underline this in both lay and professional practice, and future interventions should seek to change these wider determinants of infant feeding in addition to supporting individual families. In what way these norms, however, can be disentangled from ambitions and anxieties of being “a good mother” (Marshall, Godfrey, & Renfrew, 2007; May, 2008), seem challenging, and would clearly also need to address health professionals not only the parents.

### Strengths and Limitations

This qualitative study could explore experiences of participants of a healthy formula-feeding trial by inviting some of the participating mothers to reflect on their own practices and potential change as well as the delivery of the trial. Taking a wider theoretical perspective beyond testing the trial intervention’s psychological framing helped to understand underlying mechanisms that disrupted the delivery such as experiences of stigma and self-reliance. Some of the trial’s challenges also turned into opportunities for this qualitative study. The recruitment into the trial of participants with older infants challenged their adherence to the intervention guidance, as discussed above; however, for this qualitative study, it enabled us to compare their experiences of infant formula-feeding before and after entering the trial.

There were also limitations to this study. The overall backdrop of addressing and investigating a stigmatized practice—infant formula-feeding—also shaped this qualitative study. The interviewers felt as cautious as the intervention facilitators during their interactions to sensitively probe into transgressions and challenges of the new mothers. Research in this area is still scarce, and longer term research designs with time to develop rapport and trust could provide more in-depth insights into feeding practices. More sophisticated qualitative research designs that go beyond, for example, small interview or focus groups studies could greatly enhance the understanding of participants’ experiences (Asiodu, Waters, Dailey, & Lyndon, 2017), and inform interventions both in their planning and evaluation phase. In particular, such sophisticated qualitative research designs could better contribute to accounting for complexity in complex interventions, exploring in-depth the target behavior(s) (here: infant feeding, mothering, and so forth) and target population (here: women of varied backgrounds, social support, experiences with health professionals, and stigma).

## Conclusion

This qualitative study could explore in depth the trial participants’ and facilitators’ experiences and reflections on participating in a behavioral intervention, and contribute to a growing understanding of how social factors can shape behavioral interventions in unanticipated ways. Lessons learned from this study are informative for future interventions. Addressing a highly stigmatized social practice, the intervention facilitators seemed to have inhabited a role of providing pastoral care, and delivering the program, therefore, included a degree of permissiveness. Moreover, the mothers’ practices seem to have been shaped by implicit and explicit societal norms that relate an infant’s health with weight gain, which stood in conflict with the premise of the intervention guidance whose message of “excess weight” seemed attenuated. The psychological behavior change framework employed in the intervention could not address this complexity adequately. Most notably, the most positive outcome of the trial participation for the mothers, probably not captured in the trial’s quantitative outcome measures but a central finding in this qualitative study, was the personal and nonjudgmental support they received.

This most appreciated element of the trial might clearly be difficult to scale-up into routine practice. However, our participants’ experiences clearly highlighted the need to receive nonjudgmental information and support right from the birth of their infants to develop healthy ways of formula-feeding. Formula-feeding mothers should not be ignored if excessive formula-milk amounts are recognized to contribute to childhood obesity, and new mothers should certainly not be stigmatized at a time of great anxiety, uncertainty, and vulnerability. Arguably, this is a controversial proposition and some might argue that providing formula-feeding support might discouraged breastfeeding. Yet, in other public health fields such as the prevention of sexually transmitted diseases and teenage pregnancy, the public health stance favors harm reduction, the provision of advice and condoms, without fearing to discourage abstinence (Underhill, Montgomery, & Operario, 2007). A solution could be to provide support and information material more generally for “combination feeding” that would avoid singling out and therefore stigmatizing particular feeding practices (Asiodu et al., 2017). How to challenge societal norms that promote excess weight gain in infants seems an equally challenging task; that the *Baby-Milk Trial* managed to achieve changed feeding practices at least indicates an opportunity to do so.

## Supplemental Material

Suupplementary_Material – Supplemental material for Toward Understanding How Social Factors Shaped a Behavioral Intervention on Healthier Infant Formula-Feeding Click here for additional data file. Supplemental material, Suupplementary_Material for Toward Understanding How Social Factors Shaped a Behavioral Intervention on Healthier Infant Formula-Feeding by Cornelia Guell, Fiona Whittle, Ken K. Ong and Rajalakshmi Lakshman in Qualitative Health Research
