# Supplementary material for: Toward Understanding How Social Factors Shaped a Behavioral Intervention on Healthier Infant Formula-Feeding
Source: Qual Health Res. 2018 Mar 21;28(8):1320–9. doi: 10.1177/1049732318764386 (PMC6038024; doi:10.1177/1049732318764386)
Supplement: Suupplementary_Material – Supplemental material for Toward Understanding How Social Factors Shaped a Behavioral Intervention on Healthier Infant Formula-Feeding [file Supplementary_Material.pdf]

Online Only Supplement 1 – Interview Guides

Participant interview guide: Exploring infant feeding practices and experience of participation in an infant feeding trial

Before interview starts:

- Introduce myself, stress that
- taking part in this additional qualitative study is voluntary, and that
- I have not been involved in the design of the intervention.

Recap intervention:

Research suggests that many mothers who formula-feed their babies would like to have more information and support. The intervention was set up to understand these needs, in order to improve the advice for future generations.

Introduce aims of my research project: *I want to know about*

1. *how you experienced feeding your baby*
2. *how you made decisions about how to feed your baby*
3. *I want speak to people with a wide range of experiences and opinions on infant feeding,*
   I'm interested in your honest views- there are "no right or wrong answers".
4. *I'd also like know about your experiences of taking part in the intervention.*
5. *I am not testing you on how well you completed the intervention, but I am interested in how well taking intervention fitted in with your personal circumstances (or not!).*

You may have answered some of the questions that I will be going through in the questionnaires or have talked with the *nurses* but I would like to explore these further in the interview. Unlike the discussions you had with the *nurses*, I will not be giving you any advice or information but I want you to tell me about **your** experiences. ....

Interview starts:
Switch on tape recorder
Record participant STUDY ID number; ask for consent, record if anyone else is present

Semi-structured questions in bold, *potential prompts/probes in Italics*

--

WARMING UP/ BUILDING RAPPORT QUESTION

a) **How did you find out about the Intervention?**
b) **Why did you decide to take part?**
c) **Can you tell me a bit about your family?** *Married/co habiting, first child?, other*

children?

d) **Childcare set up?** *Commercial/regular, relatives?*

1 OBJECTIVE 1: EXPLORING EXPERIENCES OF INFANT FEEDING INCLUDING FORMULA MILK & INTRODUCTION OF SOLID FOOD

1.1 Can you tell me your feeding 'history/journey' with this child (first 6 months)?
- *what age did baby first have formula, combination/mixed feeding or purely formula milk, change over*

1.2 Can you tell me your about your experience of feeding your baby?

1.3 What are the most important factors that affect your decisions about feeding your baby?

1.4 Who is involved in the decision making process?

1.5 What do you consider to be the positive aspects (benefits) associated with of formula feeding? *What was easy?*

1.6 Were there any negative aspects (disadvantages, difficulties) of formula feeding? *What was difficult?*

1.7 Have you sought out information, advice or support on how you feed your baby (milk feeds)?
- *If yes, where/who from?*
- *If yes, why?*

1.8 How could your experience of feeding formula milk to your baby have been improved?

1.9 Anything else you'd like to add about milk feeding?

1.10 Can you tell me about your experience with weaning/introducing solid food to your baby?

1.11 How could your experience of weaning/introducing solid food to your baby have been improved?

2 OBJECTIVE 2: TO EXPLORE PARTICIPANTS' EXPERIENCE OF TAKING PART IN INTERVENTION

2.1 Can you tell me what you know about the group you were allocated to?

2.1.1 **INTERVENTION group:** How did you feel about finding out that you would be helped to follow a NEW feeding programme?

2.1.2 **CONTROL group:** How did you feel about finding out that you would be given STANDARD advice about bottle-feeding and weaning?”

Questions for Intervention and Control group

2.2 How did you find participating in the intervention?

2.3 Was it similar or different to what you expected?

2.4 What were the best parts of the intervention?

2.5 What were the worst parts of the intervention?

2.6 What do you think the intervention was trying to achieve?

2.7 Did it make you do anything differently?

2.8 What did you feel about the additional measurements taken during the intervention

Questions for INTERVENTION group only

2.9 Did you try to follow the feeding recommendations/ personalised feeding plan?

2.9.1 *If yes,* What was trying to follow the feeding recommendations like?

2.9.2 *If no,* What were the main reasons for not following the plan?

2.9.3 What programme elements did you like/dislike? *Why/why not?*

- *leaflets, stickers*
- *face-to-face discussions, telephone discussions*

Questions for CONTROL group only

2.10 Did you pick up any information about the intervention during your participation in the intervention? (if yes explore how, from whom, to what extent).

2.11 What feeding recommendations did you follow?

3 FINAL THOUGHTS / RECOMMENDATIONS

3.1 Do you have anything else that you would like to add about your experience of taking part in INTERVENTION?

3.2 Do you have any recommendations to improve future infant feeding programmes- especially where these programmes have to be delivered within limited resources (e.g. within NHS)?

3.3 What would be the most useful aspects?

3.4 Do you have any recommendations regarding infant feeding advice/information?

3.5 What advice would you give to any first time mums about feeding?

3.6 Do you have anything else that you would like to add about your experience of feeding your baby?

Thank you very much!

Facilitator interview guide: Exploring infant feeding practices and experience of participation in an infant feeding trial

Before interview:
Introduce myself and project aims:

- qualitative study using individual semi-structured interview to explore facilitators' attitudes, beliefs and experiences of delivering the intervention.
- participation in this qualitative study is voluntary, with refusal to take part having no effect on employment status,
- data will be anonymous, interested in the facilitators' honest views and experiences.

Ask facilitators to sign the consent form.

Interview starts:
Switch on tape recorder
Record participant Facilitator ID number; ask for consent to record, record if anyone else is present.

Semi-structured questions in bold, *potential prompts/probes in Italics*

Objective 1 Beliefs and experiences of delivering the intervention

What were your initial thoughts when you found out about the idea/content of the programme?

How did you find delivering the programme?

Which aspects of the intervention do you think worked well?

In your view what were the main challenges?

Which aspects of the intervention do you think could be improved?

How would you suggest to improve recruitment/retention in a potential scaled-down 'real-world', pragmatic roll out through the Healthy Child Programme delivered by Health Visitors?

Based on your experiences of delivering the programme how do you feel about the idea/content of intervention now?

Have you learnt anything about feeding babies that you didn't know before?

Objective 2 Perception of participants' experience

How do you think mothers in the Intervention group experienced taking part in the intervention?

-explore ability to follow the intervention protocols

How do you think mothers in the Control group experienced taking part in the intervention?

-explore possibility of contamination

(If not already covered) How do you think mothers in the Intervention group felt about your feeding advice?

-explore ability to follow intervention

Do you think the information you provided had an effect on the participant's decision making or in any other way?

Do you have any recommendations regarding providing infant feeding advice to mothers *(what information, who by, what format, when?)*

Do you have anything else that you would like to add about your experience of advising mothers on feeding their babies?

Background section 1: About your work history
Did you have any previous experience of working as a trial facilitator / research nurse before working the intervention? If yes, briefly describe your experience, please.
Did you have any previous work experience of giving infant feeding advice before working the intervention? If yes, briefly describe your experience, please.

Background section 2: About your infant feeding experience
Do you have children? How many children do you have? What year(s) were they born?
Did you breastfeed any of your children?

If yes, how many of your children did you breastfeed?

If yes, what is the longest duration that you breastfed any of your children?

Did you formula feed any of your children?

If yes, how many of your children did you formula feed? How long for?

Did you do any combined feeding/mixed feeding (formula& breastfeeding) with any of your children?
Do you think your experiences of feeding your own children have influenced how you deliver the intervention?

Thank you for your participation.

Online Only Supplement 2: Code Book

	Coding tag	Description
1	Reason	Reason for formula feeding
2	CMidW	Contact with Midwives (Hospital)
3	CHP	Contact with health professionals (Post-hospital)
4	CF	Contact with Facilitator
5	Conf Adv.	Conflicting advice
6	No Adv.	No advice
7	Prev Own	Previous experience – own
8	Prev Other	Previous experience – others
9	InfoF	Information source – formula
10	InfoW	Information source - weaning
11	Stigma	Stigma (both bottle and breast feeding)
12	GLTin	Guidelines tin
13	GLBM	Guidelines Baby Milk - Baby Milk amounts or any of the programme elements i.e. giving water, looking for cues from baby
14	GLTrans	Guidelines transgression – when guidelines were not followed due to baby wellbeing/response etc.
15	MonAm	Monitor amount (Include overfeeding)
16	MonGrow	Monitor growth
17	Support	Family/Peer/Social support
18	Father	Involvement of Father
19	Neg	Negatives: Practicalities, cost etc.
20	Pos	Positives: Lifestyle, sharing feeding/bonding, practicalities, routine
21	1stMum	First time mum - lack of confidence / anxiety
22	ForBaby	Best for baby

23	Self-rel	Self-reliance
24	Study	Study participation – recruitment, logistics
25	Contam	Contamination
26	StudyImp	Study impact – did it make a difference, outcomes, understanding of aims

-- - Practical/Social
-- - Advice/Experience of Professionals
-- - Information sources
-- - Study participation
-- - Guidelines
-- - Stigma
